# Numerical Modeling and Optimization Design of Embedded Rubber Waterstops in Tunnel Lining

**DOI:** 10.3390/polym17030421

**Published:** 2025-02-05

**Authors:** Xuan Fan, Hailin Wang, Chaoran Xie, Mingfeng Lei, Chenjie Gong

**Affiliations:** 1 School of Civil Engineering, Central South University, Changsha 410075, China; csu_fanxuan@csu.edu.cn (X.F.); csu_xcr@csu.edu.cn (C.X.); mingdfenglei@csu.edu.cn (M.L.); 2 Hunan Provincial Communications Planning, Survey & Design Institute Co., Ltd., Changsha 410200, China; hailin_wang@126.com

**Keywords:** tunnel lining, embedded rubber waterstop, numerical modeling, deformation performance, optimization design

## Abstract

Tunnel water leakage is a common issue. Embedded rubber waterstops are crucial in ensuring the waterproofing performance of mountain tunnels. The deformation performance of a rubber waterstop directly impacts its effectiveness, with structural parameters playing a key role. This study employs numerical simulation methods to quantitatively assess the impact of structural parameters—such as the central hole, ribs, and flanges—on the deformation performance of waterstops. The parametric analysis reveals significant variations in how different structural components affect the deformation performance, as indicated by the defined deformation stress influence rate. Specifically, the deformation performance of the embedded waterstop under tensile, compression, and settlement deformations shows a correlation with factors such as the ratio of the central hole opening rate to thickness and the inner and outer diameters. Additionally, an optimization analysis, taking both economic and performance factors into account, was conducted on 16 types of waterstops with different central hole parameters, from which the optimal waterstop was selected. This research provides a scientific basis for enhancing the deformation performance of waterstops and optimizing their structure.

## 1. Introduction

**Figure 1:**
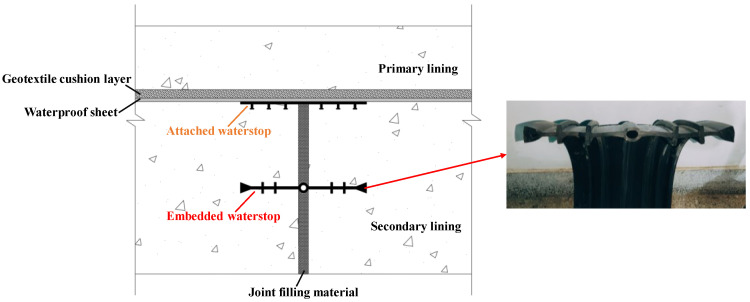
Schematic diagram of tunnel joint waterproofing system.

Leakage is a common defect in tunnels, with many tunnels, both under construction and completed, exhibiting varying degrees of water leakage [1]. This issue not only complicates the construction process but also significantly undermines the safety and durability of the tunnel, potentially posing serious risks to the safety of users and their property [2]. As such, tunnel waterproofing has become a critical concern requiring immediate and focused attention. The waterproofing system of mountain tunnels typically consists of concrete self-waterproofing, waterproof layers, and joint waterproofing (Figure 1). Any failure in one part of the system can lead to the failure of the entire waterproofing system, making it essential to ensure the waterproofing capability of each component [3,4].

Several researchers have conducted in-depth studies on the components of the waterproofing system, yielding valuable insights. In the waterproofing system, the self-waterproofing of concrete largely depends on its impermeability. In recent years, many studies have focused on enhancing the impermeability of concrete by incorporating fibers [5], while others have proposed applying polymer coatings to the surface of concrete to further improve its waterproofing performance [6,7,8]. The waterproof layer, located between the primary and secondary linings of tunnel, serves as a critical waterproof barrier, typically composed of a geotextile layer combined with a geomembrane or waterproof sheet [9]. Regarding the geomembrane, which plays a key role in the waterproof layer, Luciani et al. [10] designed an accelerated aging device to study the durability of plasticized polyvinyl chloride (PVC) geomembranes and proposed a method to estimate their service life. Addressing the lack of standardized quantitative testing for the airtightness of weld scars, Qin et al. [11] proposed quantitative standards for testing the airtightness of weld scars at the overlap joints of waterproof sheets through model testing and numerical simulations and verified through field experiments that the proposed standards met the required airtightness quality control. While research on the waterproof layer and concrete self-waterproofing performance is relatively abundant, studies focusing on the waterstops at the joints remain relatively scarce and require further investigation.

The joints are the weak points in the secondary lining and represent a critical component in the overall waterproofing performance of the mountain tunnel. The waterproofing performance of the joints primarily relies on the waterstop, which is therefore the key to ensuring the effectiveness of the entire mountain tunnel’s waterproof system. Waterstops can be classified into rubber waterstops, PVC waterstops, and composite material waterstops, among others [12]. The most common type is the rubber waterstop, which is favored due to the excellent elasticity, aging resistance, corrosion resistance, and waterproof performance of rubber as a polymer material [13]. Two main types of waterstops are used at the joints: embedded waterstops and attached waterstops. As a commonly used joint waterproofing material, the embedded waterstop is integrated into the concrete structure, working in conjunction with the concrete to form an effective waterproof barrier. Its primary function is to maintain the seal at the joint by stretching, compressing, or settling as the concrete on both sides of the joint deforms, thereby preventing water from infiltrating the structure through the joint [14]. Through the appropriate design and selection of structural parameters for the waterstop, its ability to adapt to deformation and its waterproof performance can be effectively enhanced, thereby extending the service life of the structure.

Some researchers have conducted valuable studies on waterstops, focusing primarily on experimental research and numerical simulation. In terms of experimental research, Cho et al. [15] proposed a method using a special double-sided adhesive tape to enhance the bonding between the waterstop and the concrete interface. Waterproofing tests have shown that the use of double-sided tape significantly improves the waterproofing performance of the waterstop. Wu et al. [16] suggested that the bonding behavior between the waterstop and concrete could be modeled using a bilinear cohesive force model and conducted experiments to measure the tensile and shear strength at the interface between the rubber and the waterstop. These experimental results were further validated through numerical simulations. In terms of numerical simulation research, Wang et al. [17] used numerical simulation methods to study the impact of waterstop hardness and the bond strength between the waterstop and concrete on its deformation and waterproofing performance. They also proposed an optimization method for the bond strength between the waterstop and concrete. Wu et al. [18] proposed that the waterproofing function of the waterstop primarily relies on two mechanisms: compression sealing and adhesive water blocking. They also categorized the interface between the waterstop and the concrete into three regions: the bonding failure zone, bonding damage zone, and bonding zone. Zhai et al. [19] established a numerical model for the waterstop and concrete based on the Euler–Lagrange coupling method. The interaction between water and the waterstop during the seepage process was analyzed, and the influence of water pressure and material type on the waterproofing performance of the waterstop was also investigated.

Currently, research on rubber waterstops primarily focuses on their deformation adaptability and bonding performance with concrete. With the development of new materials and structures, the design and performance studies of rubber waterstops have gradually expanded, particularly in improving their overall functionality and durability, where some progress has been made. However, there is still insufficient research on the structural parameters of the various components of the waterstop and their interactions, and the existing studies lack systematic and in-depth analysis. Furthermore, there is no unified standard or specification for the design of rubber waterstops; the design process largely relies on engineering experience and practical application feedback. This experience-based design approach lacks scientific foundation and repeatability, making it challenging to meet the increasingly complex engineering requirements and environmental conditions. The absence of clear design standards and parametric analysis also hinders the optimization and assurance of waterstop performance across different engineering applications. The stresses on waterproof materials are complex in tunnel lining, as factors such as groundwater, temperature, and vehicle loads can cause significant deformation of the waterproofing materials. Therefore, it is necessary to study the structural parameters and deformation performance of rubber waterstops. By reasonably selecting and designing the structural parameters, the deformation and waterproof performance of waterstops can be significantly improved.

This study investigates the embedded rubber waterstop using numerical simulation methods to explore the impact of various structural parameters on its deformation resistance. By defining and analyzing the deformation stress impact factor, the study quantitatively evaluates how different structural parameters influence the waterstop’s ability to withstand deformation. Special focus is placed on the central hole structure, which plays a key role during deformation, and the study clarifies how variations in the inner diameter, outer diameter, and hole ratio affect the deformation performance of the waterstop. Additionally, this study conducts an optimization analysis on waterstops with different central hole parameters, considering both performance improvement and economic efficiency. Through in-depth analysis and optimization of the structural parameters of the embedded rubber waterstop, this research provides a scientific foundation for improving the structural rationality and performance of the waterstop while ensuring its economic viability.

## 2. The Establishment of the Rubber Waterstop–Concrete Numerical Model

Before establishing the numerical model for the concrete–waterstop system, it is essential to determine the parameters for the concrete, the rubber waterstop, and the bond between the concrete and waterstop. Additionally, to assess the failure of the waterstop, it is necessary to define the failure criteria for the waterstop. In the numerical simulation of the concrete–waterstop model, the main failure mechanisms include the debonding at the interface between the concrete and waterstop and the deformation failure of the waterstop itself. Because concrete does not suffer damage due to the deformation of the waterstop, it can be modeled using a linear elastic approach. Therefore, concrete with a strength grade of C30 was considered, with an elastic modulus of 30 GPa and a Poisson’s ratio of 0.2 [20].

### 2.1. Constitutive Model of the Rubber Waterstop

Rubber is a hyperelastic material with characteristics of both geometric and material nonlinearity [21]. When conducting numerical simulations for rubber waterstops, it is necessary to define the constitutive equation for the rubber material [22,23,24]. In existing studies, commonly used models include the Mooney–Rivlin model [25], Yeoh model [26], and Ogden model [27], based on the theory of continuum mechanics, as well as the Arruda-Boyce model [28], Gent model [29], and neo-Hookean model, which are based on thermodynamic statistical theory [30]. Among these, the Mooney–Rivlin model (hereafter referred to as the M–R model) is the simplest and most widely used and is more computationally stable compared with other models. Therefore, this study employs the M–R model as the constitutive model for rubber, with the strain energy expression being given by:

(1) $$w=C_{10}\left(I_{1}-3\right)+C_{01}(I_{2}-3)$$

In the expression, the strain energy w is a linear function of $I_{1}$ and $I_{2}$, where $I_{1}$ and $I_{2}$ are the first and second invariants of strain, respectively. The parameters $C_{10}$ and $C_{01}$ are the material mechanical property constants, which are the parameters that need to be determined.

**Figure 2:**
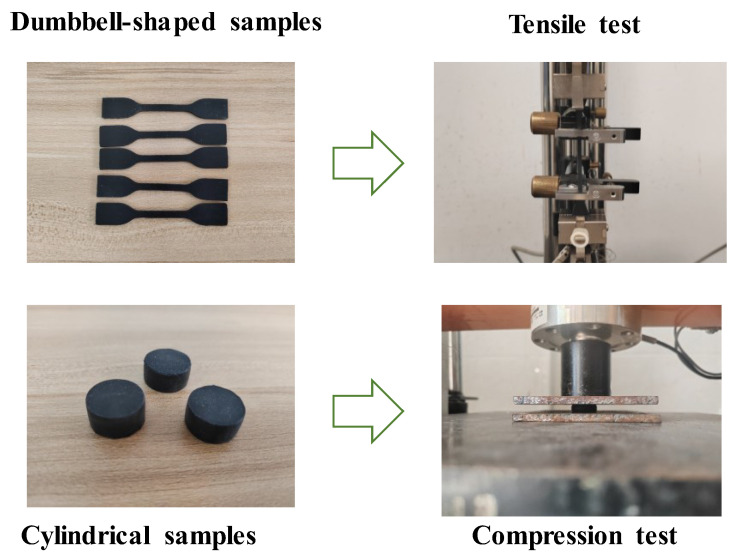
Tensile and compression tests of rubber standard samples.

The constitutive parameters of the Mooney–Rivlin (M–R) model are generally determined by fitting the results of rubber tensile tests. However, considering that local compressive stresses may occur in the rubber waterstop during the simulation process, the M–R model parameters in this study were fitted using both tensile and compressive test results of the rubber. Tensile and compressive tests were conducted using rubber dumbbell-shaped and cylindrical samples (Figure 2), respectively. And the tensile and compression tests were conducted in accordance with the standards [31]. The results of these tests were then fitted to obtain the generalized M–R constitutive model for the rubber. The resulting constitutive parameters are as follows: $C_{10}$ = 0.593 MPa, $C_{01}$ = 0.314 MPa.

### 2.2. Contact Model

The bond between the rubber waterstop and concrete interface can be simulated using a bilinear cohesive model [32,33,34]. The key parameters for this interface bond include the tangential bond strength, normal bond strength, tangential stiffness, normal stiffness, and plastic displacement. Existing studies have shown that these key parameters can be obtained through bonding tests between rubber materials and cement mortar specimens [18]. In this study, the tangential bond strength and normal bond strength are chosen as 0.151 MPa and 0.386 MPa, respectively, while the tangential stiffness and normal stiffness are set to 1510 and 3860, respectively. The plastic displacement is taken as 0.0009 mm.

### 2.3. Failure Criteria for the Rubber Waterstop

Existing studies suggest that when subjected to tensile forces, synthetic rubber and other polymers should not exceed a stress level of 20% to ensure a service life that meets the 100-year requirement for underground structures [35,36]. According to standards, the tensile strength of rubber waterstops should not be less than 10 MPa. Therefore, a value of 2 MPa can be considered as the critical stress level for the failure of the rubber waterstop.

### 2.4. Element Types and Boundary Conditions

**Figure 3:**
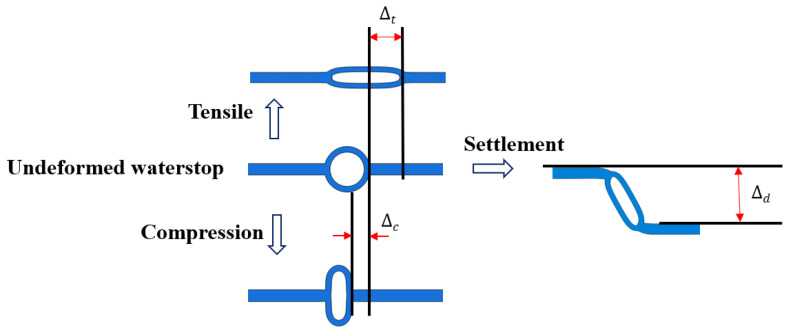
Schematic diagram of tensile, compression, and settlement deformation.

The numerical model consists of two parts: the rubber waterstop and the concrete. Fixed constraints are applied to one side of the concrete adjacent to the rubber waterstop, while displacement constraints are applied to the other side. The fixed side remains stationary, and the displacement constraints on the other side allow for horizontal and vertical movements to simulate the tensile, compression, and settlement displacements of the concrete at the expansion joint. The tensile, compression, and settlement deformations of the waterstop are shown in Figure 3. The applied deformation values are 10 mm in tension, 10 mm in compression, and 30 mm in settlement. The rubber waterstop and the concrete internal bonding interface are modeled with normal hard contact, while the tangential behavior is governed by a penalty function method, with bonding being specified as cohesive contact. Normal hard contact is also applied between the outer surface of the central hole of the rubber waterstop and the concrete, with the tangential behavior modeled using the penalty function method. Self-contact is implemented within the central hole of the rubber waterstop. The concrete is modeled using CPE4 elements, while the rubber waterstop is modeled using CPE4RH elements.

**Figure 4:**
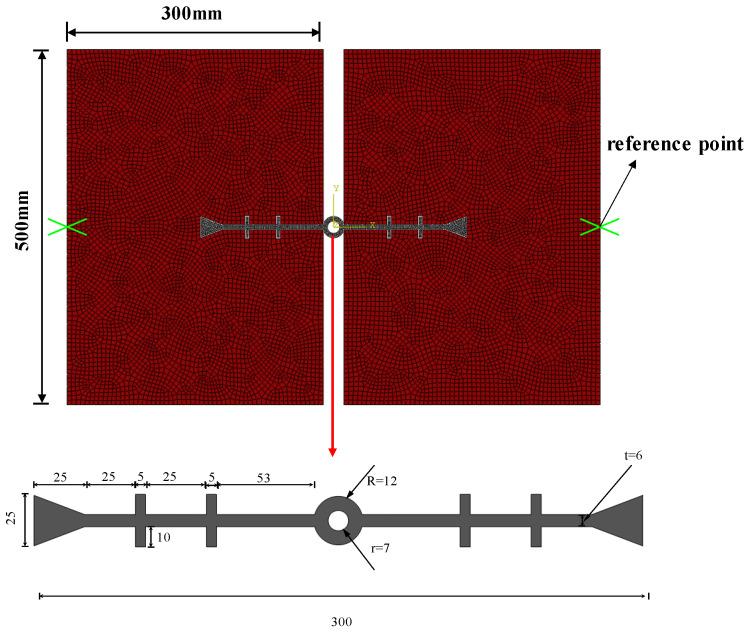
Numerical model of the concrete-embedded waterstop and the dimension of the waterstop.

The load–displacement conditions are applied by fixing the left-side concrete and imposing tensile, compressive, and settlement displacements at the reference point of the right-side concrete. The width of the rubber waterstop is 300 mm, with specific parameters being illustrated in Figure 4. The finite element model of the rubber waterstop and concrete is shown in Figure 4, where the dimensions of the concrete on both sides of the model are 500 mm and 300 mm, respectively.

### 2.5. Numerical Model and Simulation Conditions

In practical engineering, rubber waterstops experience deformation under tension, compression, and settlement. The structural parameters influencing the deformation of rubber waterstops include the size of the central hole, flange dimensions, rib size, and tail shape. To clarify the stress adaptability of the main structural components of the rubber waterstop under various deformation conditions, a quantitative analysis was conducted on the central hole, ribs, and flanges, as shown in Table 1.

### 2.6. Tensile Test of the Watersop and Verification of the Numerical Model

#### 2.6.1. Tensile Test of the Waterstop

**Figure 5:**
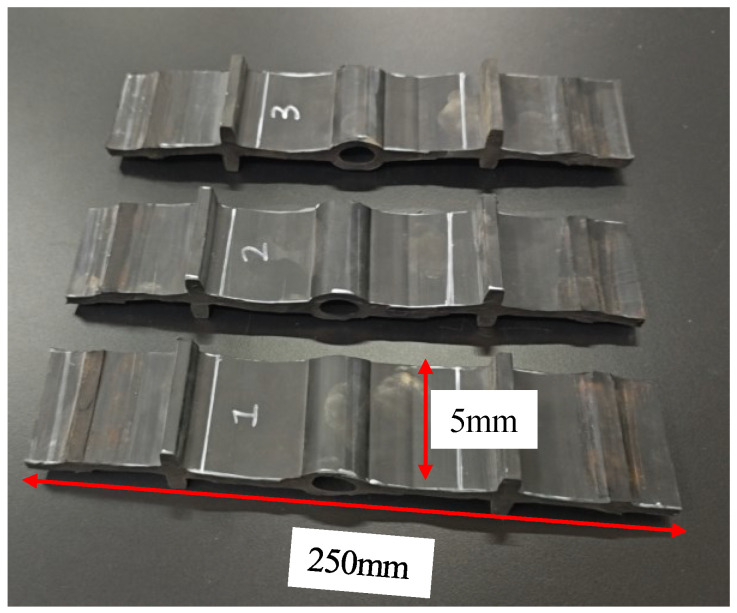
Water stop sample in tensile test.

In order to investigate the deformation and mechanical behavior of the waterstop under tensile deformation, a tensile test was conducted. The rubber waterstop used in the test was identical to the one in the numerical model. Three samples (shown in Figure 5), each with a width of 5 mm, were cut from different locations along a roll of waterstop to minimize the influence of material inhomogeneity on the experimental results. Considering that deformation primarily occurs between the first ribs on either side of the central hole of the waterstop and that the irregularity of the tail wings may affect the clamping effectiveness of the testing fixture, the tail wings and the first rib adjacent to the tail wings were removed during the preparation of the samples. The tensile test was terminated when the distance between the first ribs on either side of the central hole of the waterstop reached 20 mm, and the results were obtained as the average of the three samples.

**Figure 6:**
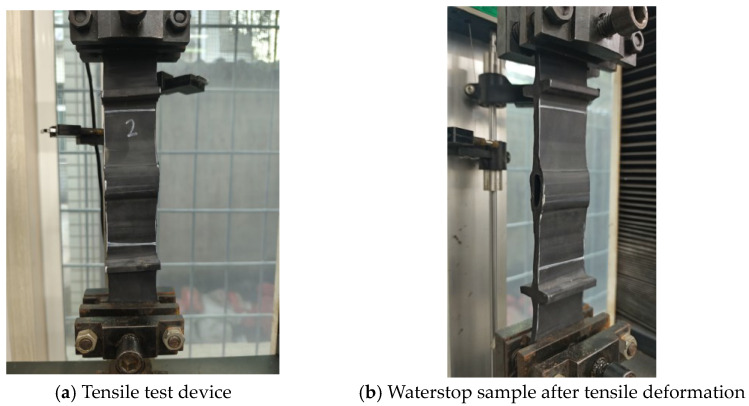
Tensile test device and test results.

The tensile test was conducted using a universal testing machine, with the test setup being shown in Figure 6a. During the test, the deformation of the waterstop was assessed by measuring the distance between two white lines. The maximum range of the force sensor was 1 kN, and the loading rate for the tensile test was 10 mm/min.

The results of the tensile test are presented in Table 2. When the tensile deformation at the first rib on both sides of the central hole reached 20 mm, the forces for samples 1–3 were 0.252 kN, 0.250 kN, and 0.244 kN, respectively, with corresponding overall tensile deformations of 38.63 mm, 41.60 mm, and 40.26 mm. The average values of force and overall deformation for the three samples were 0.249 kN and 40.16 mm, respectively. The tensile test results for the three samples were consistent with the average values, indicating that the variation in sample location had minimal impact on the results and that the rubber waterstop used exhibited excellent processing quality and uniform material properties. Additionally, as shown in Figure 6b, it can be observed that the deformation of the waterstop under a 20 mm tensile deformation is primarily concentrated between the first ribs on both sides of the waterstop. However, with continued loading, as the deformation of the rubber near the central hole becomes more significant, the entire waterstop undergoes considerable deformation.

#### 2.6.2. Verification of the Numerical Model

**Figure 7:**
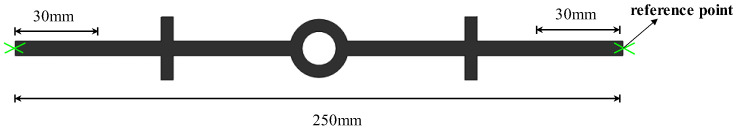
Tensile model of the waterstop sample.

In order to validate the established numerical model, a numerical model of the rubber waterstop sample used in tensile test was developed using ABAQUS/Standard, as shown in Figure 7. For the material parameters of the model, the Mooney–Rivlin model was used for simulation, with the constitutive model parameters being derived from tensile and compression tests of standard rubber samples. A fully fixed constraint was applied on one side of the waterstop, while to compare and validate the model with the experimental tensile results, a displacement corresponding to the average overall deformation of the three samples (40.16 mm) was applied to the other side. The gripping length of the fixtures at both ends of the waterstop is 30 mm. The displacement was applied at the reference point.

The reaction force at both ends of the waterstop were extracted from the numerical results, with the calculated reaction force being 0.212 kN. This value deviates by 14.9% from the measured value obtained in the tensile test. The discrepancy may be attributed to an overestimation of the deformation on both sides of the waterstop during the experimental measurements. However, as the difference is relatively small and less than the measured value, it can be concluded that the numerical simulation results are on the conservative side. Therefore, it is reasonable to consider the use of the rubber constitutive model parameters obtained from tensile and compression tests for simulating the deformation of the rubber waterstop.

## 3. Numerical Analysis of Structural Parameters

### 3.1. Parameters of the Central Hole

In the analysis of the central hole parameters, three different cases were considered: a fixed inner diameter of the central hole with varying outer diameters, fixed outer diameter with varying inner diameters, and fixed thickness with varying inner diameters. The specific conditions for each case are shown in Table 3. A total of 16 different forms of waterstops with varying central hole parameters are considered, with each number corresponding to a different form of the waterstop. Hole opening ratio λ is defined as:

(2) $$\lambda =\frac{A_{1}}{A_{2}}$$

**Figure 8:**
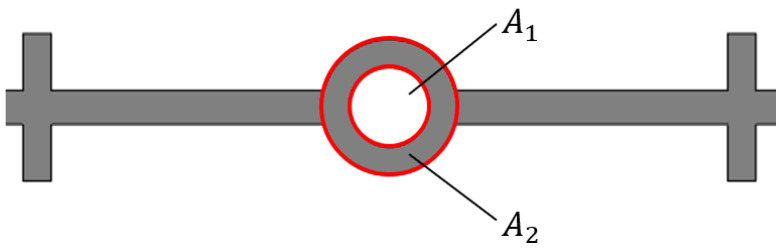
Schematic diagram of the opening area of the central hole.

$A_{1}$ and $A_{2}$ represent the opening area and total area of the central hole, respectively, which are shown in Figure 8.

**Figure 9:**
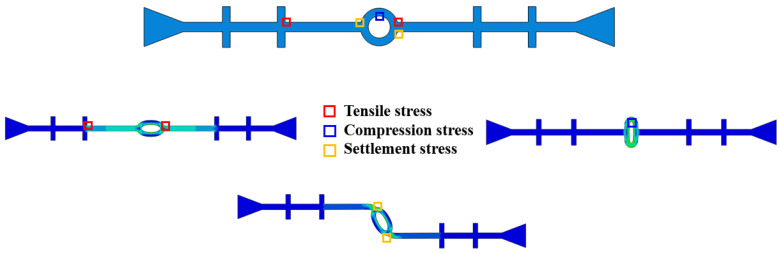
The locations of the maximum stress under tensile, compression, and settlement deformation.

In the numerical simulation of the tensile, compression, and settlement deformation of the rubber waterstop, the deformation performance of the waterstop is assessed by extracting the maximum deformation stress. Figure 9 illustrates the approximate distribution of deformation stress and the locations where the maximum deformation stress occurs under the three types of deformation. In Figure 9, it can be observed that the maximum tensile deformation stress occurs at the junction of the first rib near the central hole and the flange, as well as at the junction of the outer side of the central hole and the flange. The maximum compression deformation stress is located at the upper and lower ends of the inner side of the central hole, while the maximum settlement deformation stress is found at the junction of the outer side of the central hole and the flange.

#### 3.1.1. Fixed Inner Diameter of the Central Hole

**Figure 10:**
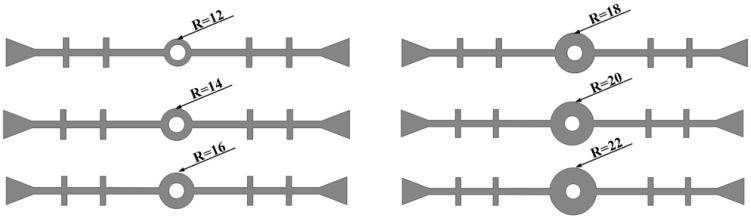
Fixed inner diameter of the central hole with varying outer diameters (units: mm).

The inner diameter of the central hole is kept constant (*r* = 7 mm), and six different conditions with varying outer diameters of the central hole are considered to establish a plane strain finite element model (as shown in Figure 10). Three types of deformation—a tensile deformation of 20 mm, compression deformation of 10 mm, and settlement deformation of 30 mm—are applied, without considering concrete damage during deformation.

**Figure 11:**
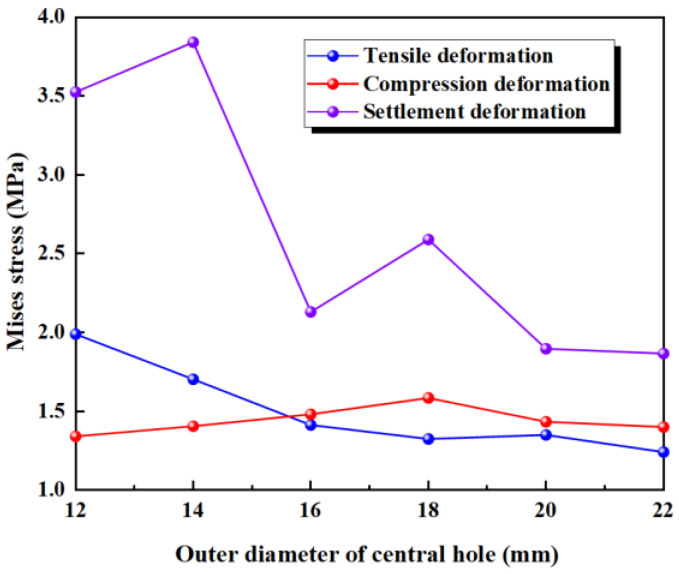
The stress of tensile, compression, and settlement deformation with different outer diameters.

The results of the numerical simulation are shown in Figure 11, where the maximum deformation stresses during tensile, compression, and settlement deformations are extracted. The deformation stress for waterstops with different outer diameters are presented in Figure 11. In Figure 11, with the fixed inner diameter of the central hole, as the outer diameter increases, the deformation stress shows a decreasing trend. Furthermore, for waterstops with different outer diameters, the deformation stress never exceeds the critical stress of 2 MPa, except when *R* = 12 mm, where it approaches 2 MPa.

**Figure 12:**
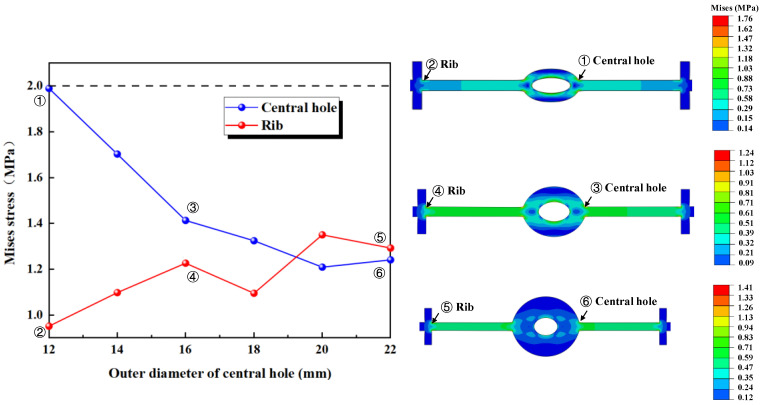
The distribution pattern of maximum stress in tensile deformation.

Figure 12 illustrates the maximum stress at the junctions of the first rib near the central hole and the flange, and the junction of the outer side of the central hole and the flange, under tensile deformation. It can be observed that when *R* is less than 18 mm, the stress at the junction of the outer side of the central hole and the flange is higher, while when *R* exceeds 18 mm, the stress at the junction of the first rib near the central hole and the flange exceeds the stress at the junction of the outer side of the central hole and the flange. The results of the numerical simulation indicates that, with the fixed inner diameter of the central hole, as the outer diameter increases, the thickness of the central hole increases, leading to a decrease in the deformation performance of the central hole. Consequently, the applied tensile deformation shifts from being concentrated around the central hole to being concentrated at the flange between the central hole and the first rib. As a result, the stress at the central hole decreases, while the stress at the rib increases.

Under a compression deformation of 10 mm, the regions where the maximum deformation stress may occur are at the upper and lower ends of the inner side of the central hole. With the fixed inner diameter of the central hole, as the outer diameter increases, the deformation stress first increases slightly and then decreases slightly, with little overall change. Additionally, for waterstops with different outer diameters, the deformation stress never exceeds the critical stress of 2 MPa.

Under a settlement deformation of 30 mm, the region where the maximum deformation stress may occur is at the junction of the outer side of the central hole and the flange. With the fixed inner diameter of the central hole, as the outer diameter increases, the deformation stress initially exceeds 3.5 MPa for smaller outer diameters but decreases to around 2.5 MPa for larger outer diameters, showing a significant overall change. When *R* is less than 18 mm, the maximum stress exceeds the critical stress of 2 MPa, whereas when *R* exceeds 18 mm, the maximum stress remains below the critical stress.

#### 3.1.2. Fixed Outer Diameter of the Central Hole

**Figure 13:**
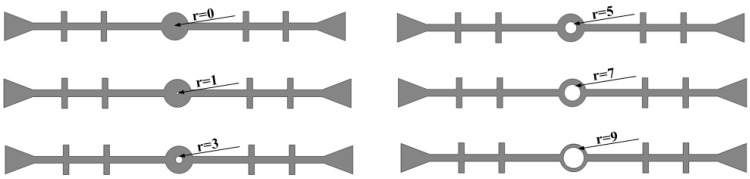
Fixed outer diameter of the central hole with varying inner diameters (units: mm).

The outer diameter of the waterstop is kept constant (*R* = 12 mm) while gradually increasing the inner diameter, which results in a decrease in the thickness of the central hole (Figure 13). We calculate and analyze the conditions for the following six different inner diameters, keeping all other model settings consistent with previous sections.

**Figure 14:**
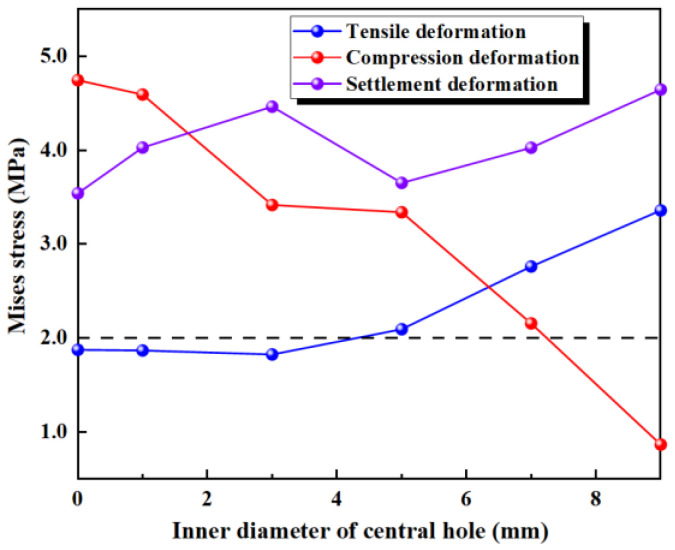
The stress of tensile, compression, and settlement deformation with different inner diameters.

The results of the numerical simulation are shown in Figure 14, where the maximum deformation stress of the waterstop with a fixed inner diameter and varying outer diameters of the central hole under applied tensile, compression, and settlement deformations are extracted. In Figure 14, when the outer diameter of the waterstop belt remains constant, as the inner diameter of the central hole increases, the deformation stress first remains unchanged and then gradually increases. Moreover, when the outer diameter is less than 5 mm, the maximum stress is below the critical stress of 2 MPa, while for the outer diameter of greater than 5 mm, the maximum stress exceeds the critical stress.

**Figure 15:**
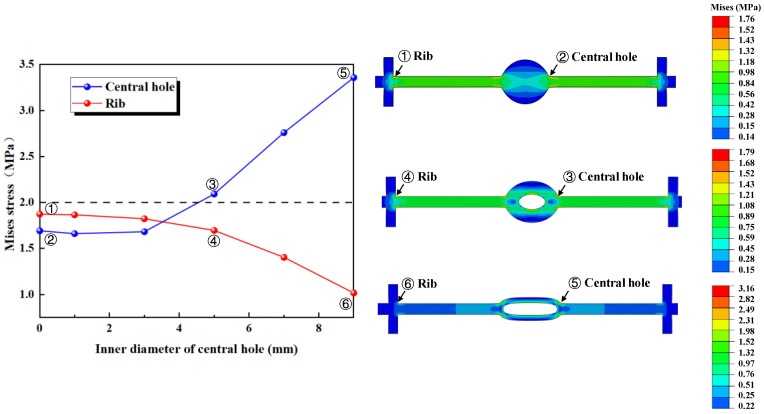
The distribution pattern of maximum stress in tensile deformation.

Figure 15 illustrates the maximum stress at the junctions of the first rib near the central hole and the flange, and the junction of the outer side of the central hole and the flange, under tensile deformation. When *R* is less than 5 mm, the stress at the junction of the first rib near the central hole and the flange is higher, while when *R* is greater than 5 mm, the stress at the junction of the outer side of the central hole and the flange is higher. With the outer diameter of the central hole kept constant, as the inner diameter increases, the thickness of the central hole decreases, and the deformation performance of the central hole increases. As a result, the applied tensile deformation concentrates at the central hole, causing the stress to increase, while the stress at the rib decreases.

Under a compression deformation of 10 mm, the locations where the maximum deformation stress may occur in the waterstop are at the upper and lower ends of the inner side of the central hole. When the outer diameter of the waterstop remains constant, as the inner diameter of the central hole increases, the deformation stress first decreases, then stabilizes, and finally decreases again. Overall, the deformation stress continuously decreases, and except for the case where the inner diameter is 9 mm, the deformation stress for other smaller inner diameters exceeds the critical stress of 2 MPa.

Under a settlement deformation of 30 mm, the locations where the maximum deformation stress may occur in the waterstop are at the junction of the outer side of the central hole and the flange. With the outer diameter of the central hole kept constant, as the inner diameter increases, the deformation stress first increases, then decreases, and then increases again. For all waterstops with different inner diameters, the deformation stress exceeds the critical stress of 2 MPa and generally stays around a stress level of 4 MPa. Moreover, changing the inner diameter does not result in a significant reduction in the stress level.

#### 3.1.3. Fixed Thickness of the Central Hole

**Figure 16:**
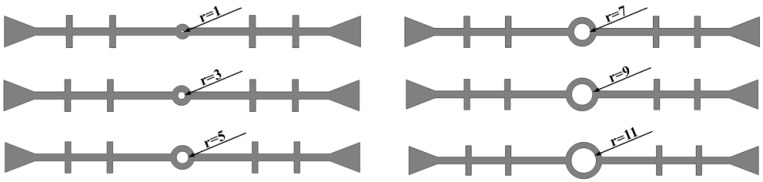
Fixed thickness of the central hole with varying inner diameters (units: mm).

The thickness of the central hole in the waterstop is kept constant (*h* = 5 mm), while the inner diameter of the waterstop gradually increases. Because the thickness remains unchanged, the outer diameter also increases accordingly (Figure 16). The following six different inner diameters are selected for the numerical analysis, keeping all other model settings consistent with previous sections.

**Figure 17:**
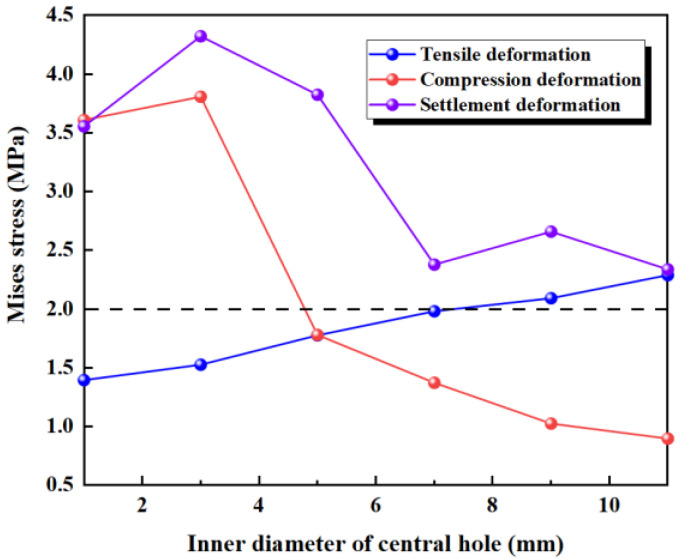
The stress of tensile, compression, and settlement deformation with fixed thickness of the central hole.

The results of the numerical simulation are shown in Figure 17, where the maximum deformation stress of the waterstop with fixed thickness and varying inner diameters of the central hole under applied tensile, compression, and settlement deformations are extracted. In Figure 17, it can be observed that when the thickness of the central hole remains constant, as the inner diameter increases, the deformation stress shows an increasing trend. Moreover, when the outer diameter (*R*) is less than 7 mm, the maximum stress is below the critical stress, whereas for *R* greater than 7 mm, the maximum stress exceeds the critical stress.

**Figure 18:**
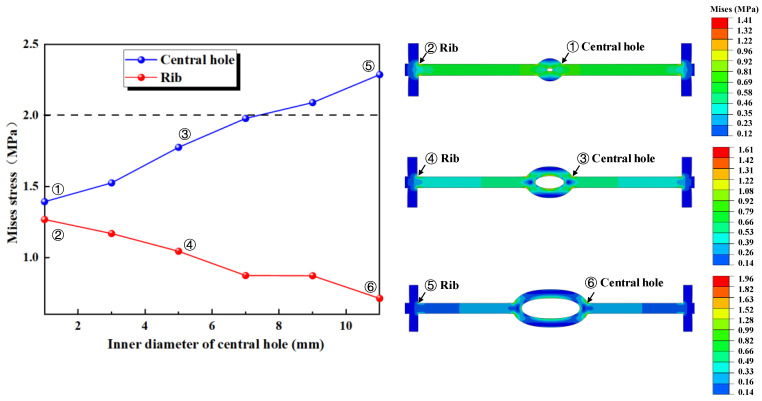
The distribution pattern of maximum stress in tensile deformation.

Figure 18 illustrates the maximum stress at the junctions of the first rib near the central hole and the flange, and the junction of the outer side of the central hole and the flange, under tensile deformation. As the inner diameter of the central hole increases, the stress at the junction of the first rib near the central hole and the flange gradually decreases, while the stress at the junction of the outer side of the central hole and the flange increases. With the thickness of the central hole being kept constant, as the inner diameter increases, the outer diameter of the central hole also increases. As a result, the deformation performance of the flange between the first rib and the central hole decreases, and the applied tensile deformation becomes increasingly concentrated at the central hole, leading to a continuous increase in the stress at the central hole.

Under a compression deformation of 10 mm, the locations where the maximum deformation stress may occur in the waterstop are at the upper and lower ends of the inner side of the central hole. When the thickness of the central hole remains constant, as the inner diameter increases, the deformation stress first increases slightly, then continuously decreases. When *R* is less than 5 mm, the maximum stress exceeds the critical stress, while for an *R* of greater than 7 mm, the maximum stress is below the critical stress. This behavior is because when the inner diameter is small, the central hole in the waterstop bears compression deformation, resulting in stress concentration at the central hole.

Under a settlement deformation of 30 mm, the location where the maximum deformation stress may occur in the waterstop is at the junction of the outer side of the central hole and the flange. With the thickness of the central hole being kept constant, the deformation stress remains above 3.5 MPa when the outer diameter is small. As the inner diameter increases, the stress level decreases to around 2.5 MPa. Overall, there is a noticeable variation in the stress distribution. When *R* is less than 18 mm, the maximum stress exceeds the critical stress of 2 MPa; when *R* is greater than 18 mm, the maximum stress is below the critical stress.

### 3.2. Parameters of the Rib

#### 3.2.1. The Number and Placement of the Ribs

**Figure 19:**
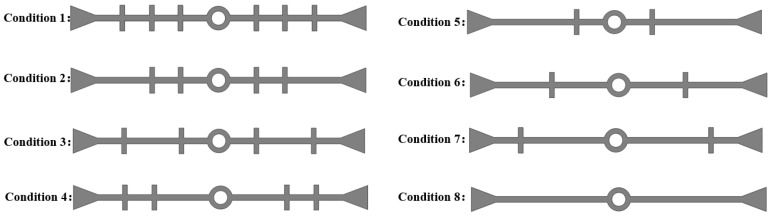
Different number and placement of ribs in the waterstops (units: mm).

We consider a waterstop with 0, 2, 4, or 6 ribs on one side of the flange, and with the ribs being symmetrically arranged; eight different conditions have been established by varying the placement of the ribs (Figure 19). All other model settings remain consistent with those outlined in previous sections.

**Figure 20:**
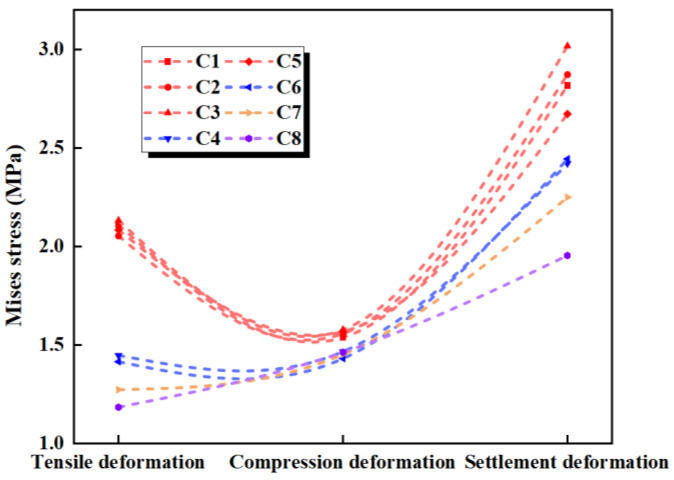
Stress of tensile, compression, and settlement deformation under different conditions.

The maximum deformation stresses for the eight different types of waterstops under applied tensile, compression, and settlement deformations were extracted and are shown in Figure 20. In Figure 20, it can be observed that under tensile deformation, the deformation stresses in the waterstop of C1, C2, C3, and C5 are very similar, while the deformation stresses in C4 and C6 are also closely aligned. Among these, the deformation stresses in C1, C2, C3, and C5 are the highest, followed by C4 and C6, and C7 has the next highest stress, with the smallest deformation stress being observed in C8. Referring to Figure 20, it can be seen that while the number and arrangement of ribs in C1, C2, C3, and C5 differ, the distance from the first rib to the central hole is the same on both sides of the hole. The same applies to C4 and C6. The tensile deformation stress does not show a significant correlation with the number and arrangement of ribs but is rather influenced by the distance from the first rib to the central hole. As this distance increases, the deformation stress tends to decrease gradually.

Under compression deformation, the deformation stresses in C1–C8 differ slightly by about 0.1 MPa. This indicates that the number and position of the ribs have minimal impact on the compression deformation stress.

Under settlement deformation, the maximum deformation stresses occur in C1, C2, C3, and C5, ranging from 2.5 to 3.0 MPa. The stresses in C4 and C6 are somewhat lower, followed by C7, with the smallest deformation stress occurring in C8. Similarly, as shown in Figure 20, the settlement deformation stress is not related to the number or position of the ribs but is primarily influenced by the distance from the first rib to the central hole on both sides of the hole. Although there is a stress difference of 0.5 MPa among the maximum stresses in C1, C2, C3, and C5, overall, as the distance from the first rib to the central hole increases, the settlement deformation stress decreases.

#### 3.2.2. The Dimensions of the Ribs

**Figure 21:**
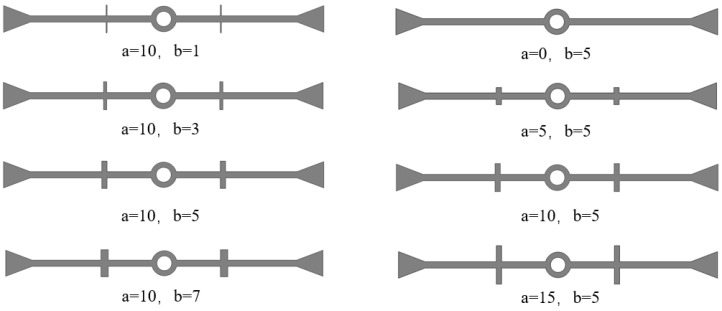
Different dimensions of ribs on the waterstops (units: mm).

The cross-section of the ribs on the waterstop is typically rectangular, with the height generally being greater than the width. In the numerical simulation, only two ribs are placed on each side of the waterstop, keeping their placement fixed. Each condition varies only the length a or the width b based on the initial dimension of the rib on the waterstop, as illustrated in Figure 21, while the other model settings remain consistent with the previous section. All other model settings remain consistent with those outlined in previous sections.

**Figure 22:**
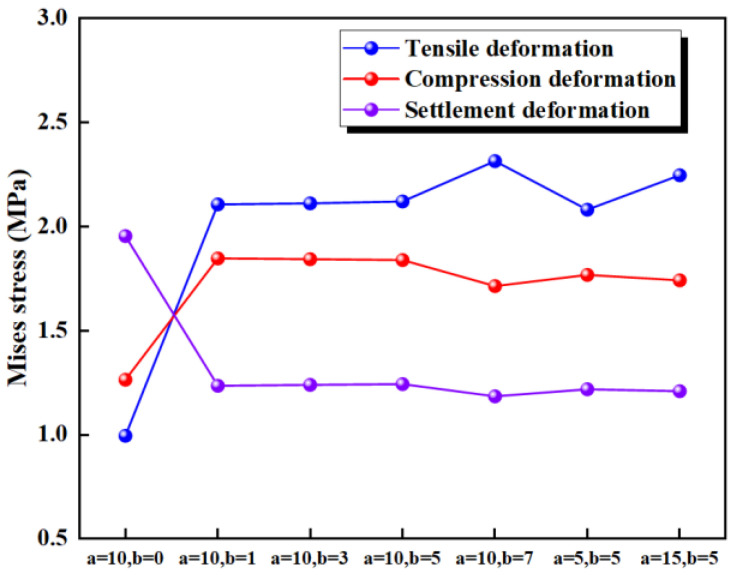
Stress of tensile, compression, and settlement deformation under different dimensions.

The maximum deformation stresses of the eight different types of waterstops under applied tensile, compression, and settlement deformations were extracted and are shown in Figure 22. Under tensile deformation, the deformation stress of the waterstop without ribs is lower than that of the ribbed waterstop. For ribbed waterstops, the rib length and width have little impact on the tensile deformation stress, and the deformation stress exceeds the critical stress in all conditions. Under compression deformation, the deformation stress of the waterstop without ribs is lower than that of the ribbed waterstop, and the deformation stress is below the critical stress. The rib length and width have minimal effect on the compression deformation stress of the ribbed waterstop. Under settlement deformation, the deformation stress of the ribbed waterstop is lower than that of the waterstop without ribs, and in both cases, the deformation stress is below the critical stress. The length and width of the ribs have little effect on the settlement deformation stress of the ribbed waterstop.

#### 3.2.3. The Distance Between the Ribs and the Center Hole

**Figure 23:**
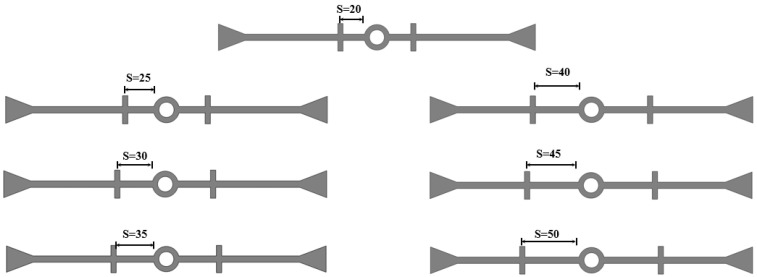
Different distance from the first rib to the central hole (units: mm).

To investigate the influence of the distance from the rib to the central hole on the deformation stress of the waterstop, seven waterstop models with rib-to-center hole distances of s = 20 mm, 25 mm, 30 mm, 35 mm, 40 mm, 45 mm, and 50 mm were designed (Figure 23). All other model settings remain consistent with those outlined in previous sections.

**Figure 24:**
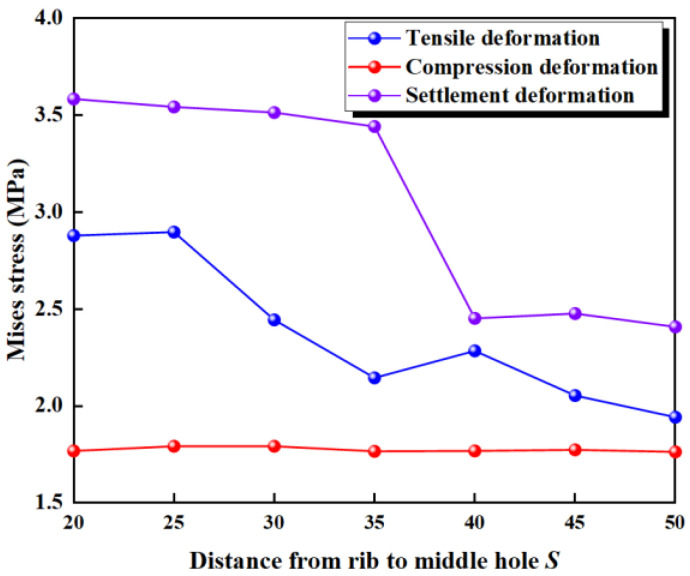
Stress of tensile, compression, and settlement deformation under different distances.

The maximum deformation stresses of seven different types of waterstop under tensile, compression, and settlement deformation were extracted and are shown in Figure 24. Under tensile deformation, the stress initially decreases with an increasing distance from the rib to the central hole, then slightly increases, and subsequently decreases again. Overall, there is a decreasing trend, although when the rib-to-hole distance reaches its maximum, the tensile deformation stress still exceeds the critical stress. Under compression deformation, the compression deformation stress remains almost unchanged as the rib-to-hole distance increases, indicating that the rib-to-hole distance has minimal impact on the compression deformation stress. In all conditions, the compression deformation stress of the waterstop is below the critical stress. Under settlement deformation, the settlement deformation stress initially remains largely unchanged with an increasing rib-to-hole distance, then sharply decreases and stabilizes, overall showing a decreasing trend. However, when the rib-to-hole distance is large, the settlement deformation stress still exceeds the critical stress.

### 3.3. Parameters of the Flange

#### 3.3.1. The Thickness of the Flange

**Figure 25:**
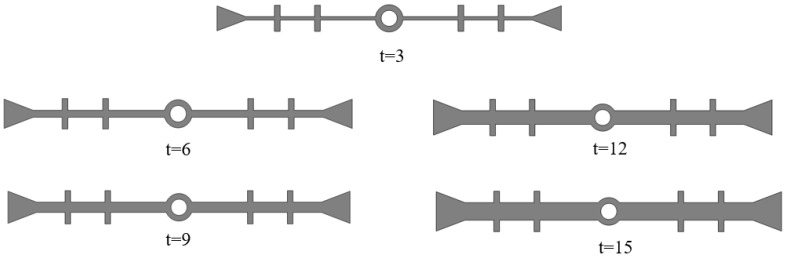
Different thickness of the flange (units: mm).

To investigate the effect of flange thickness on the deformation stress of the waterstop, five waterstop models with flange thicknesses of 3 mm, 6 mm, 9 mm, 12 mm, and 15 mm were designed (Figure 25). All other model settings remain consistent with those outlined in previous sections.

**Figure 26:**
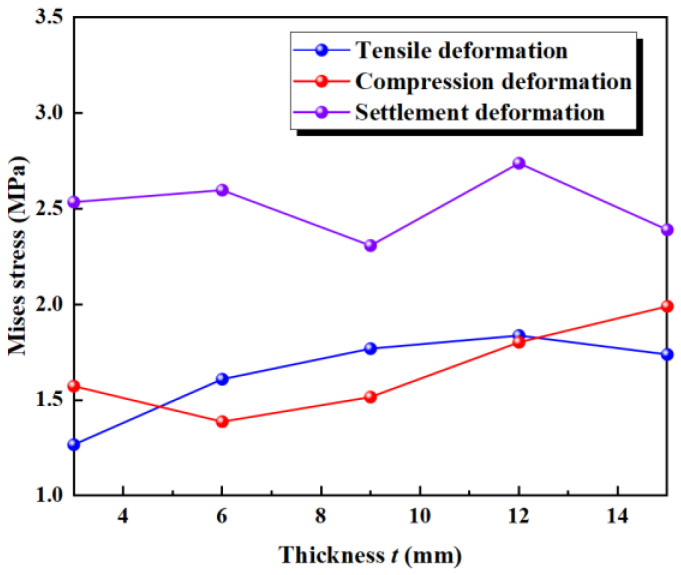
Stress of tensile, compression, and settlement deformation under different thickness.

The maximum deformation stresses of five different waterstops with varying flange thickness under tensile, compression, and settlement deformation were extracted and are shown in Figure 26. Under tensile deformation, the stress initially increases with the flange thickness, then gradually decreases, showing an overall increasing trend. When the flange thickness is large, the tensile deformation stress approaches and slightly falls below the critical stress. Under compression deformation, the stress slightly decreases with increasing flange thickness, then continues to increase, showing an overall increasing trend. When the flange thickness is large, the compression deformation stress is close to and slightly below the critical stress. Under settlement deformation, the stress first slightly decreases, then increases, and finally decreases again with increasing flange thickness. The stress level remains around 2.5 MPa, exceeding the critical stress in all conditions. The effect of flange thickness on tensile, compression, and settlement deformation stress is relatively small, as there was no obvious instance of the stress being reduced to below the critical stress.

#### 3.3.2. The Length of the Flange

Quantitative analysis of the rib placement indicates that deformation in the waterstop mainly concentrates between the first rib and the central hole. Therefore, changes in the flange length of the waterstop have little impact on its deformation and stress distribution.

From the working mechanism of the waterstop, it is understood that the flange length affects the seepage path, which in turn influences the waterproofing performance of the waterstop. Key factors influencing the seepage path include flange length, rib dimensions, and the size of the flange end. A longer flange results in a longer seepage path, and thus increasing the flange length can improve the waterproofing performance of the waterstop to a certain extent.

However, because the waterstop is placed at construction joints, it exerts a cutting effect on the concrete on both sides of the joint. The larger the flange length, the greater the area of concrete subjected to cutting, which can weaken the integrity of the concrete lining, leading to defects and vulnerable zones in the lining. This reduces the waterproofing performance at the construction joint, which contradicts the intended purpose of enhancing the waterproof performance at the joint. Therefore, when designing a waterstop, both the seepage path and the cutting effect on the concrete should be considered. The flange length should not be set excessively large or small.

#### 3.3.3. The Shape of the Flange End

Similarly, when the waterstop is ribbed, the deformation and stress are concentrated between the first ribs on both sides of the waterstop, without significantly affecting the flange end. Therefore, the shape of the flange end does not have a notable impact on the deformation and stress distribution of the waterstop.

## 4. Parametric Analysis

### 4.1. Deformation Stress Influence Rate

Through a quantitative analysis of key structural parameters such as the central hole, rib, and flange in the waterstop, it can be observed that under tensile, compression, and settlement deformations, the deformation primarily concentrates between the first rib on both sides. Furthermore, the central hole plays a crucial role in the deformation process of the waterstop.

In Section 3, “Numerical Analysis of Structural Parameters”, approximately six gradient values were designed for each parameter. However, the resulting deformation stress distribution ranges of the waterstop vary across different parameters. For some parameters, changes lead to a reduction in deformation stress from around 4 MPa to approximately 1 MPa, while for others, the stress remains between 3 and 4 MPa. This suggests that different structural parameters exert varying degrees of influence on the deformation stress of the waterstop. In practical applications, identifying which parameters have a more significant impact on the deformation stress would allow for targeted adjustments to achieve optimal results. However, existing studies do not seem to offer effective analytical indicators or methods to assess the sensitivity of these parameters. Therefore, to quantitatively analyze the impact of structural parameters on the stress experienced by the waterstop during deformation, we define a new metric: the deformation stress influence rate φ, which can be calculated by Equation (3). The maximum ($\sigma_{max}$) and minimum ($\sigma_{min}$) deformation stress are extracted from a set of different parameters of specific conditions. The deformation stress influence rate is characterized by the ratio of the difference between the maximum and minimum deformation stresses to the maximum deformation stress. A higher deformation stress influence rate indicates a more significant effect of the conditions on the deformation stress of the waterstop, implying greater sensitivity of the waterstop to these conditions. The deformation stresses for different forms of waterstops under tensile, compression, and settlement deformations are presented in Table 4.

(3) $$\varphi =\frac{\sigma_{max}-\sigma_{min}}{\sigma_{max}}$$

For waterstops with varying side thicknesses of the central hole, the deformation stress influence rates are ranked from highest to lowest as follows: settlement (51.40%), tension (37.62%), and compression (4.25%). This indicates that when the inner diameter of the central hole is at a reasonable value, settlement deformation is most significantly affected by changes in side thickness, with the maximum deformation stress approaching twice that of the minimum. Given that the maximum settlement deformation stress is 3.84 MPa, which exceeds the critical stress for the waterstop, increasing the outer diameter (and thus the side thickness) can effectively improve settlement deformation and reduce stress. The influence rates for tensile deformation stress indicate a notable effect of thickness, with the maximum deformation stress being approximately 1.5 times the minimum. In contrast, compression deformation stress is minimally affected by thickness changes when the inner diameter is fixed. When the outer diameter is constant, the influence rates for varying thicknesses rank as follows: compression (81.78%), tension (45.70%), and settlement (23.78%). Notably, under compression, the maximum stress can be five times the minimum stress, with values of 4.74 MPa and 0.86 MPa, respectively. Thus, the inner diameter significantly impacts compression deformation stress, making it a critical parameter to consider. For tension and settlement, maximum deformation stresses are approximately twice and 1.25 times the respective minimums. Under fixed thickness, the influence rates for varying inner diameters are as follows: compression (76.45%), settlement (45.94%), and tension (39.10%), with maximum stresses under compression again being around five times the minimum. It is essential to maintain the inner diameter above 5 mm to avoid excessive stress concentrations.

Variations in the number and positioning of ribs influence deformation stress as follows: tension (46.93%), settlement (35.23%), and compression (19.92%). The positioning of the first rib on either side has the most significant impact, particularly for tensile deformation, where the maximum stress is about twice the minimum. Changes in rib size influence deformation stress rates as follows: tension (57.03%), settlement (39.42%), and compression (31.58%), with tensile deformation again showing the most significant effects. When considering varying distances from the ribs to the central hole, the influence rates are tension (32.99%), settlement (32.83%), and compression (1.63%), indicating minimal impact on compression.

Finally, for waterstops with varying flange thicknesses, the deformation stress influence rates are settlement (39.51%), tension (31.06%), and compression (30.33%), with the maximum deformation stresses for settlement and tension being approximately 1.67 times and 1.33 times the respective minimum stresses.

(a: Fixed inner diameter of the central hole, b: fixed outer diameter of the central hole, c: fixed thickness of the central hole, d: number and placement of the ribs, e: dimension of the ribs, f: distance from the ribs to the central hole, g: thickness of the flange.)

**Figure 27:**
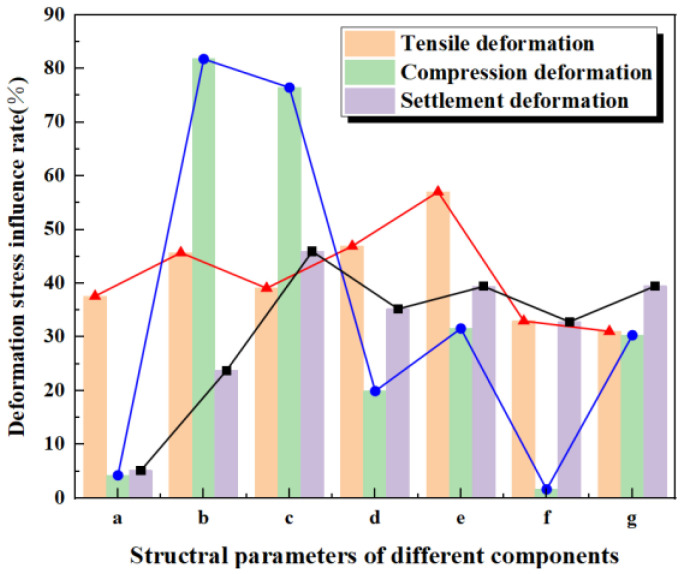
Deformation stress influence rate of different components.

As shown in Figure 27, for tensile deformation, the deformation stress influence rates across various structural parameter conditions range between 30% and 60%. The size of the ribs has the most significant impact on the deformation stress during tensile deformation, while flange thickness exhibits the least influence. In the case of compression deformation, the variation in deformation stress influence rates is considerable; specifically, the rates for different inner diameters with a fixed outer diameter and for varying thicknesses at a constant thickness are notably high, at 81.78% and 76.45%, respectively. Conversely, when the inner diameter is fixed, the influence rates for varying thicknesses and rib-to-central hole distances are much lower, at only 4.25% and 1.63%. For settlement deformation, the influence rates for all structural parameter conditions remain below 50%, with the highest rate occurring under different inner diameters for a fixed wall thickness and the lowest rate occurring with varying thicknesses at a constant inner diameter.

### 4.2. Influence of Central Hole Parameters on Deformation

A total of 16 different types of waterstops with fixed inner diameter, fixed outer diameter, and fixed thickness of the central hole were subjected to numerical simulations for tensile, compression, and settlement deformation. During the numerical simulation of these central hole parameters, it was observed that the hole ratio, inner diameter, and outer diameter of the central hole have certain influence patterns on a waterstop’s ability to withstand deformation.

**Figure 28:**
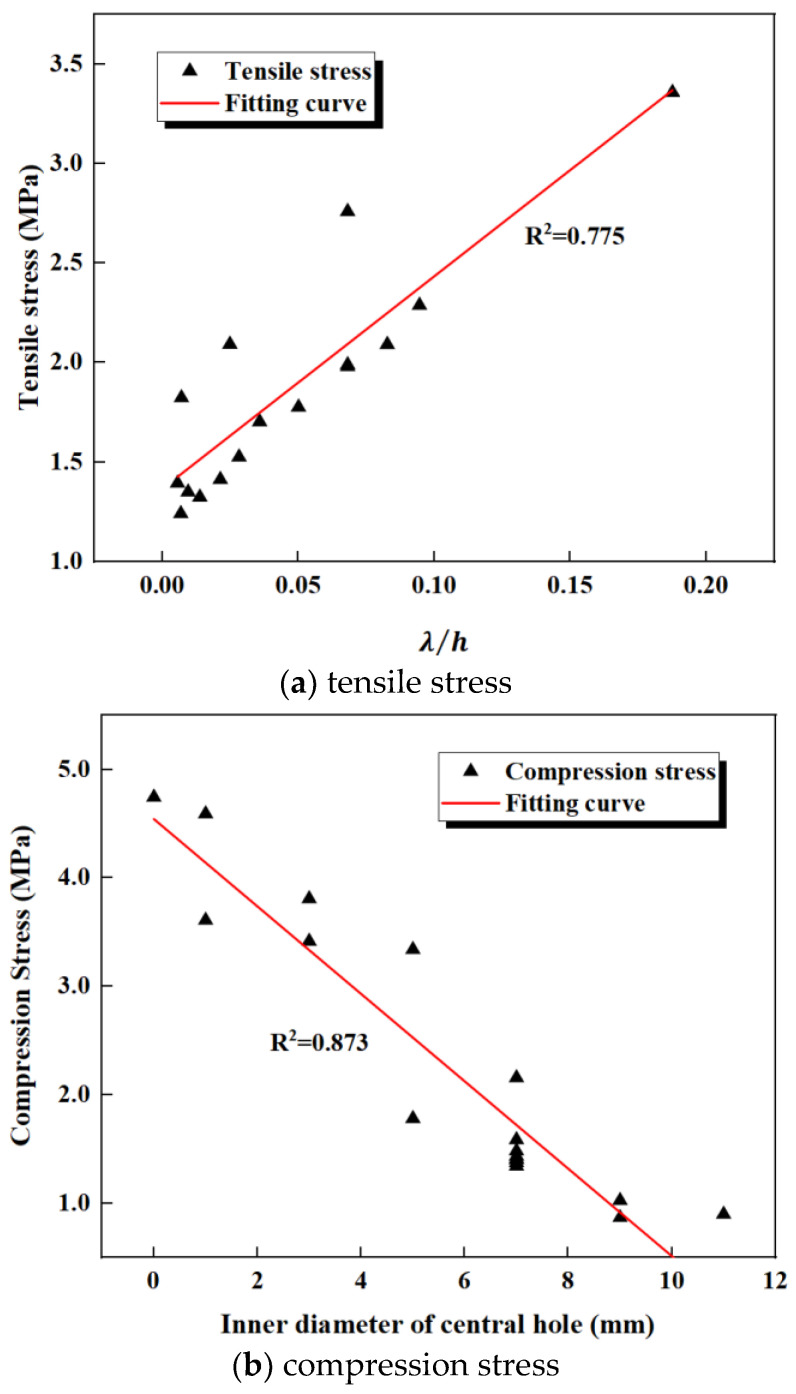

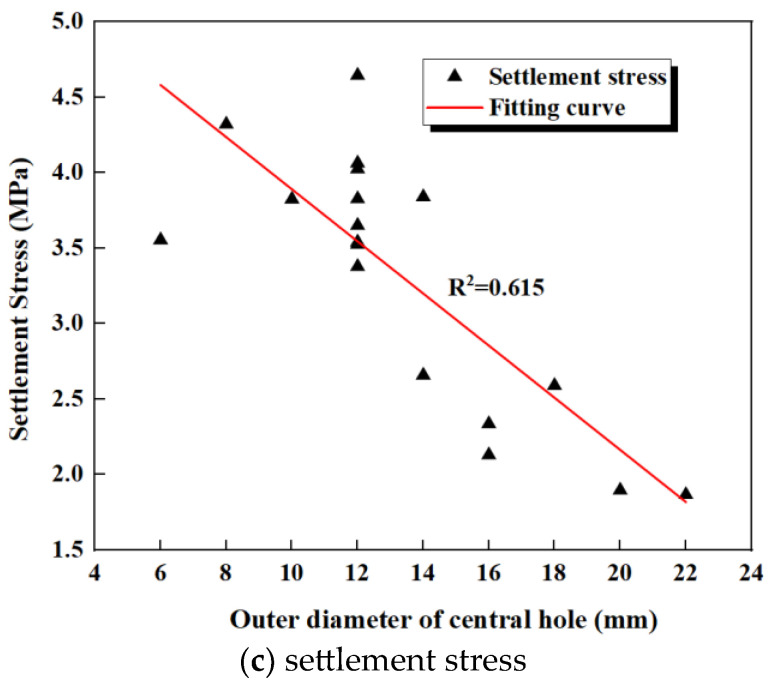
Stress and influencing factor fitting curve.

By analyzing the simulation results of the 16 different waterstops, it was found that the tensile deformation stress is proportional to the hole ratio λ and inversely proportional to the thickness h of the central hole. The relationship between the tensile deformation stress and λ/h was extracted, as shown in Figure 28a. In general, tensile deformation stress is positively correlated with λ/h, meaning that the greater the value of λ/h, the greater the tensile deformation stress.

The compression deformation stress is significantly related to the inner diameter r of the central hole but shows no significant relationship with the outer diameter, thickness, or hole ratio. The relationship between compression deformation stress and the inner diameter r of the central hole is shown in Figure 28b. It can be observed that compression deformation stress is inversely correlated with r; the larger the inner diameter r, the smaller the compression deformation stress.

The settlement deformation stress is significantly related to the outer diameter R of the central hole but shows no significant relationship with the inner diameter, thickness, or hole ratio. The relationship between settlement deformation stress and the outer diameter R of the central hole is shown in Figure 28c. It can be seen that settlement deformation stress is inversely correlated with R; the larger the outer diameter R, the smaller the settlement deformation stress.

By analyzing the relationships between λ/h, the inner diameter, outer diameter, and the tensile, compression, and settlement deformation stresses, some design guidelines for the central hole parameters of the waterstop can be provided. As shown Figure 28a, when λ/h is less than 0.05, the tensile deformation stress is lower than the critical stress, which can be achieved by adjusting the ratio of the inner and outer diameters and the thickness of the central hole. According to Figure 28b, when the inner diameter of the central hole exceeds 6 mm, the compression deformation stress is below the critical stress. Therefore, the inner diameter should be designed to be no smaller than 6 mm. Figure 28c indicates that when the outer diameter of the central hole exceeds 20 mm, the settlement deformation stress is lower than the critical stress. For waterstops with significant settlement deformation, consideration should be given to appropriately increase the outer diameter of the central hole.

### 4.3. Optimization of the Central Hole Cross-Section

In the study of the influence of central hole parameters on the deformation stress of waterstop, three cases were considered: fixed inner diameter, fixed outer diameter, and fixed thickness of the central hole. A total of 16 different forms of waterstops were numerically simulated, including cases where the maximum stress exceeded or did not exceed the critical stress. For a rubber waterstop, the cross-sectional area determines its cost, i.e., its economic feasibility. Generally, the performance of the waterstop can be improved by increasing its cross-sectional area, but this also raises the cost. Therefore, the goal of optimization is to select a relatively lower cost, which corresponds to a smaller cross-sectional area, while still achieving optimal performance with a smaller deformation stress. This is the direction for optimizing the cross-sectional design of waterstops. As the central hole significantly influences both the cross-sectional area and the deformation stress, it is necessary to analyze the economic and performance improvements of waterstops with different central hole parameters. Because of the different deformation patterns under tensile, compression and settlement deformation, the optimization of the cross-sectional area should be performed for waterstops under three different deformation conditions, respectively.

Before optimization, it is necessary to determine the parameters used to quantitatively describe the performance improvement and cost increase of waterstops. The performance improvement of waterstop means that its deformation stress is smaller when it bears deformation, so the performance improvement rate is defined as the ratio of the reduction value of deformation stress to the initial value of deformation stress. The increase in cost means that when the performance of the waterstop is improved, it is often necessary to increase the cross-sectional area of the waterstop, that is, more materials will be used, so the increase in cross-sectional area is used to characterize this increase in cost. Because the waterstop j with the largest deformation stress was considered as the form with the worst performance, it was selected as the object to be optimized, with other forms i having smaller deformation stresses. However, although other forms have smaller deformation stress and better performance, the cost may be higher, and they need to be balanced at the same time between performance improvement and cost increase. Thus, the increase rate in the cross-sectional area of waterstop i can be defined as:

(4) $$\alpha_{i}=\frac{S_{i}-S_{j}}{S_{j}}$$

$S_{i}$ and $S_{j}$ are the cross-sectional area of number i, j waterstop, i= 1, 2, 3……15, 16, j refers to the number of the waterstop form to be optimized, and i≠j.

The performance improvement rate is calculated by the following equation:

(5) $$\beta_{i}=\frac{\sigma_{j}-\sigma_{i}}{\sigma_{j}}$$

$\sigma_{i}$ and $\sigma_{j}$ are the deformation stress of number i, j waterstop, i=1, 2, 3……15, 16, j refers to the number of the waterstop form to be optimized, and i≠j.

**Figure 29:**
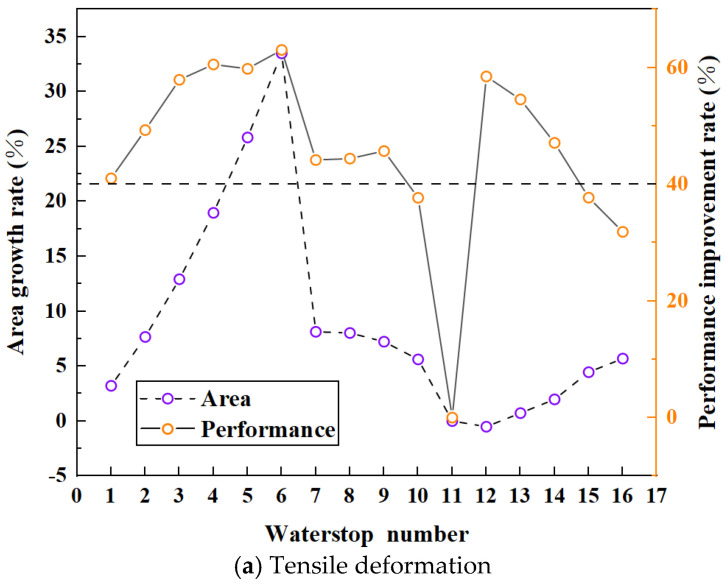

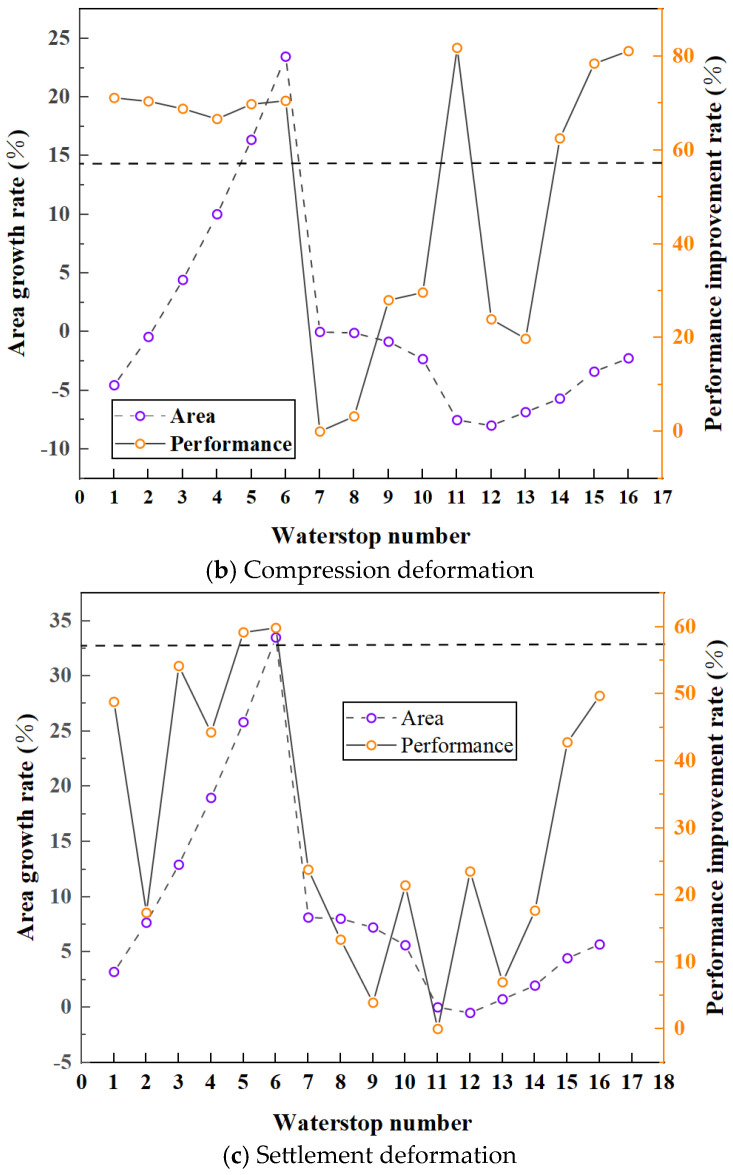
Area growth rate and performance improvement rate for tensile, compression, and settlement deformation.

The cross-sectional area increase rate and performance improvement rate for 16 different waterstop forms under tension, compression, and settlement deformation are shown in Figure 29. The goal of optimization is to maximize the performance improvement rate while minimizing the cross-sectional area increase rate, ensuring that the maximum deformation stress is below the critical stress of 2 MPa. The calculated minimum performance improvement rates for tension, compression, and settlement deformation are 40.44%, 57.85%, and 56.95%, respectively, which ensure that the maximum deformation stress of the waterstop remains below the critical stress.

For waterstop forms under tension deformation, Form 11 has the largest deformation stress, with an inner diameter of 9 mm, an outer diameter of 12 mm, and a maximum tensile deformation stress of 3.36 MPa. As shown in Figure 29a, except for Forms 10, 15, and 16, the performance improvement rates of the other waterstop forms exceed the minimum rate. Therefore, the deformation stress of 12 waterstop forms is less than the critical stress threshold. Among these 12 forms, the waterstop forms with the highest performance improvement rates are Forms 3, 4, 5, 6, 12, and 13, with performance improvement rates of around 60%. For Forms 12 and 13, the inner diameters are 1 mm and 3 mm and the outer diameters are 6 mm and 8 mm, respectively. These two forms of waterstops exhibit significant stress concentrations when subjected to compression deformation. For the other four forms of waterstops, where the performance improvement rates are similar, Form 3 has the smallest cross-sectional area increase rate at only 12.92%. Therefore, for all of the waterstop forms under tension deformation, Form 3 has the largest performance improvement rate and the smallest cross-sectional area increase rate. With an inner diameter of 7 mm and an outer diameter of 16 mm, Form 3 balances both optimal performance and cost effectiveness.

For waterstop forms under compression deformation, Form 7 has the largest deformation stress, with an inner diameter of 0 mm, an outer diameter of 12 mm, and a maximum compression deformation stress of 4.74 MPa. As shown in Figure 29b, nine waterstop forms have performance improvement rates greater than the minimum allowable value, namely Forms 1, 2, 3, 4, 5, 6, and 14, with performance improvement rates of around 70%. Forms 11, 15, and 16 have performance improvement rates of around 80%. Among them, Form 1 has a performance improvement rate of 71.1% and a cross-sectional area increase rate of −4.55%. Forms 11, 15, and 16 have performance improvement rates of 81.78%, 78.43%, and 81.10%, with cross-sectional area increase rates of −7.52%, −3.40%, and −2.25%, respectively. Due to the relatively high tensile deformation stress of Form 11, Forms 1, 15, and 16 are considered optimal for compression deformation as they offer large performance improvement rates while maintaining minimal cross-sectional area increase rates.

For waterstop forms under settlement deformation, Form 11 again has the largest deformation stress, with an inner diameter of 9 mm, an outer diameter of 12 mm, and a maximum settlement deformation stress of 4.65 MPa. In Figure 29c, only two waterstop forms have performance improvement rates greater than the minimum rate: Forms 5 and 6, with performance improvement rates of around 60%. The performance improvement rates of Forms 5 and 6 are 59.18% and 59.83%, respectively, with cross-sectional area increase rates of 25.83% and 33.50%. Therefore, under the condition that the maximum stress does not exceed the critical stress, the optimal waterstop form for settlement deformation is Form 5, as it provides the highest performance improvement rate and the smallest cross-sectional area increase rate.

Among the 16 waterstop forms, only Forms 5 and 6 satisfy the condition that the deformation stress remains below the critical stress for tension, compression, and settlement deformations. The performance improvement rates of Form 5 for tension, compression, and settlement are 59.81%, 69.79%, and 59.18%, respectively, with cross-sectional area increase rates of 25.83%, 16.37%, and 25.83%. The performance improvement rates of Form 6 for tension, compression, and settlement are 63.05%, 70.50%, and 59.83%, with cross-sectional area increase rates of 33.50%, 23.46%, and 33.50%, respectively. While the performance improvement rates of Forms 5 and 6 are relatively close, Form 5 has a smaller cross-sectional area increase rate. Therefore, among the 16 waterstop forms, Form 5 is the optimal waterstop form.

## 5. Conclusions

This study employs numerical simulation methods to investigate the influence of structural parameters of embedded waterstops on their deformation resistance performance. The influence of various structural parameters on the deformation performance of embedded waterstops was analyzed, and the extent of their influence on the deformation performance was evaluated. The relationship between the central hole parameters and the deformation performance of the waterstop was specifically discussed. Finally, an optimization analysis was conducted for different types of waterstops. This study offers valuable insights into the deformation performance of embedded waterstops and provides a scientific foundation for enhancing their structural rationality and optimizing structural parameters. The main conclusions are as follows:

1. A quantitative analysis of the central hole, ribs, and flanges of embedded waterstops was conducted to reveal the influence patterns of each structural component on the deformation performance of the waterstops. The deformation of the waterstops is concentrated between the first ribs on both sides, and the deformation performance of the central hole largely determines the overall deformation of the waterstops. Other parts primarily function to bond with the concrete and extend the seepage path.

2. The impact of different structural components on the deformation performance of embedded waterstops was analyzed by introducing the deformation stress influence rate. The results show that the influence of each structural component on deformation performance varies under different types of deformation. The size of the ribs, the inner diameter at a fixed outer diameter of the central hole, and the outer diameter at a fixed thickness of the central hole are identified as the key structural parameters influencing the tensile, compression, and settlement deformation performance of the embedded waterstops, respectively, with influence rates of 57.03%, 76.45%, and 51.40%.

3. The stress of embedded waterstops with sixteen different central hole parameters under tensile, compression, and settlement deformation conditions was analyzed. The results indicate that the tensile deformation stress is positively correlated with the ratio of the central hole opening rate to thickness, and when the ratio is less than 0.05, the tensile stress is less than the critical value; the compression deformation stress is negatively correlated with the inner diameter of the central hole, dropping below the critical value when the diameter exceeds 20 mm; the settlement deformation stress is negatively correlated with the outer diameter of the central hole, falling below the critical value when the diameter exceeds 20 mm.

4. By extracting the cross-sectional area and deformation stress of the embedded waterstops with sixteen different central hole parameters, the area increase rate and performance improvement rate were defined. The relationship between the increase in cross-sectional area and performance improvement was analyzed, and the economic and performance aspects of the embedded waterstops were optimized. Considering three deformation conditions and the constraint that the deformation stress must not exceed the critical stress, only the Form 5 waterstop satisfies all the requirements. And its performance improvement rate under tensile, compression, and settlement deformation is 59.81%, 69.79%, and 59.18%, respectively, with a cost increase of 59.81%, 69.79%, and 59.18%, respectively, which is more cost-effective than other forms of waterstops. Therefore, after comprehensive optimization, the Form 5 waterstop is the optimal design among the sixteen alternatives.

## Figures and Tables

**Table 1:** Simulation conditions.

Waterstop Structure	Structural Parameters	Simulated Working Condition	Deformation
Central hole	Fixed inner diameter r (r = 7 mm)	Outer diameter R (mm): 12, 14, 16, 18, 20, 22	Tensile deformation, compression deformation, settlement deformation
	Fixed outer diameter R (R = 12 mm)	Inner diameter r (mm): 0, 1, 3, 5, 7, 9	
	Fixed thickness h (h = 5 mm)	Inner diameter r (mm): 1, 3, 5, 7, 9, 11	
Rib	Number and position	1 or 2 or 3 ribs, with different positions	
	Dimensions (*a*, *b*)	a = 0, 5, 10, 15, b = 1, 3, 5, 7	
	Distance to the central hole (S/mm)	20, 25, 30, 35, 40, 45, 50 mm	
Flange	Thickness (t/mm)	3, 6, 9, 12, 15	

**Table 2:** Tensile test results.

Number of the Sample	1	2	3
Force when stretching 20 mm (kN)	0.252	0.250	0.244
Overall tensile deformation (mm)	38.63	41.60	40.26

**Table 3:** The parameters of the 16 types of waterstops.

	Number	**Outer Diameter** R	**Hole Opening Ratio** λ	Cross-Sectional Area (mm^2^)
Fixed inner diameter r (*r* = 7 mm)	1	12	0.34	3229.3
	2	14	0.25	3368.58
	3	16	0.191	3532.98
	4	18	0.151	3722.5
	5	20	0.122	3937.14
	6	22	0.101	4176.9
	Number	Inner diameter r	Hole opening ratio λ	Cross-sectional area (mm^2^)
Fixed outer diameter R (R = 12 mm)	7	0	0	3383.16
	8	1	0.007	3380.02
	9	3	0.063	3354.9
	10	5	0.174	3304.66
	1	7	0.34	3229.3
	11	9	0.563	3128.82
	Number	Inner diameter r	Hole opening ratio λ	Cross-sectional area (mm^2^)
Fixed thickness of central hole h (h=5 mm)	12	1	0.028	3112.9
	13	3	0.141	3151.7
	14	5	0.25	3190.5
	1	7	0.34	3229.3
	15	9	0.413	3268.1
	16	11	0.473	3306.9

**Table 4:** Deformation stress influence rate.

	Tensile Deformation	Compression Deformation	Settlement Deformation
Fixed inner diameter of the central hole	37.62%	4.25%	51.40%
Fixed outer diameter of the central hole	45.70%	81.78%	23.78%
Fixed thickness of the central hole	39.10%	76.45%	45.94%
The number and placement of ribs	46.93%	19.92%	35.23%
The dimension of the rib	57.03%	31.58%	39.42%
The distance from the central hole to the rib	32.99%	1.63%	32.83%
The thickness of the flange	31.06%	30.33%	39.51%

## Data Availability

The raw data supporting the conclusions of this article will be made available by the authors on request.
